# The gut microbiota metabolite trimethylamine-*N*-oxide in children with β-thalassemia: potential implication for iron-induced renal tubular dysfunction

**DOI:** 10.1038/s41390-024-03639-w

**Published:** 2024-10-25

**Authors:** Asmaa A. Ghalwash, Rehab M. El-Gohary, Doaa El Amrousy, Lamia M. Morad, Shaima S. Kassem, Islam Ibrahim Hegab, Asmaa H. Okasha

**Affiliations:** 1 Medical Biochemistry Department, Faculty of Medicine, Tanta University, Tanta, Egypt; 2 Pediatric Department, Faculty of Medicine, Tanta University, Tanta, Egypt; 3 Clinical Pathology Department, Faculty of Medicine, Tanta University, Tanta, Egypt; 4 Physiology Department, Faculty of Medicine, Tanta University, Tanta, Egypt

## Abstract

**Background:**

Renal tubular dysfunction is common in transfusion-dependent β thalassemia (β-TM). Iron overload, chronic anemia, and hypoxia are precipitating factors for renal insult. However, gut microbiota engagement in the renal insult has not been explored. Our work aimed to assess the potential link between iron overload, gut leakage/dysbiosis, and kidney dysfunction in these children.

**Methods:**

We enrolled 40 children with β-TM and 40 healthy controls. Gut leakage/dysbiosis biomarkers (trimethylamine-*N*-oxide [TMAO] and fecal short-chain fatty acids [SCFAs]), oxidative stress and inflammatory biomarkers, TMAO-regulated proteins such as serum sirtuin 1 (S.SIRT1) and serum high mobility box group-1 (S.HMGB1), and tubular dysfunction biomarkers were assessed. Correlations and regression analysis were performed to assess the relation between different parameters.

**Results:**

Iron overload, redox imbalance, and generalized inflammation were evident in children with β-TM. Renal tubular dysfunction biomarkers and S.TMAO were significantly elevated in the patient group. Furthermore, fecal SCFAs were significantly lower with upregulation of the investigated genes in the patient group. The correlation studies affirmed the close relationship between circulating ferritin, TMAO, and renal dysfunction and strongly implicated SIRT1/HMGB1 axis in TMAO action.

**Conclusions:**

Gut dysbiosis may have a role in the pathogenesis of renal injury in children with β-TM.

**Impact:**

- Renal tubular dysfunction is a prominent health issue in β thalassemia major (β-TM). Iron overload, chronic anemia, and hypoxia are known precipitating factors. However, gut microbiota engagement in renal insult in these patients has not yet been explored.
- We aimed to assess potential link between iron overload, gut leakage/dysbiosis, and kidney dysfunction in β-TM children and to highlight the SIRT1/HMGB1 axis, a signal motivated by the gut microbiota-dependent metabolite trimethylamine-*N*-oxide (TMAO), involvement in such insults.
- We found that gut leakage/dysbiosis may have a role in kidney dysfunction in β-TM children by exacerbating the iron-motivated oxidative stress, inflammation, ferroptosis, and modulating SIRT1/HMGB1 axis.

## Introduction

Inherited hemoglobin (Hb) disorders are the most frequent human monogenic diseases, among which those affecting the β-globin gene are the most clinically significant.^1^ β Thalassemia is instigated by a spectrum of mutations that hinder β-globin chain biosynthesis with a proportionate accumulation of unstable α-globin hemichromes.^2^ Based upon the mutation zygosity, β-Thalassemia is sub-categorized into transfusion-dependent beta-thalassemia major (β-TM), non-transfusion-dependent beta-thalassemia intermedia, and thalassemia minor.^3^

The prognosis of β-TM has improved owing to the standard therapeutics in use, including blood transfusions and iron-chelating agents. Frequent blood transfusions inevitably result in iron overload, provoking life-threatening multi-organ dysfunctions, including cardio-pulmonary disorders, endocrinal deficits, liver dysfunction, and renal impairment.^4^ Iron overload causes increased oxidative stress (OS), inflammation, ferroptosis, and dysregulated immune cell function.^5^

Renal dysfunction is the fourth most common β-TM-associated morbidity.^4,6^ Iron overload, chronic anemia, enduring hypoxia, and even some chelators are involved in the pathophysiology of β-TM-associated renal insult.^7^

The gut microbiome is profoundly recognized to exert far-reaching effects on the host physiology, immune response, and metabolism. Gut dysbiosis elects biometabolites that trigger changes in intestinal permeability and dysregulate distinct signaling pathways, causing numerous pathological malfunctions.^8^ Iron overload could disrupt the intestinal tight junction, as formerly described in an experimental rat model, ending in gut leakage.^9^ However, it is not entirely clear how frequent blood transfusion-induced iron overload and gut dysbiosis are related. Moreover, whether the gut dysbiosis is implicated in β-TM-related renal insults or not is still unclear.

Trimethylamine-*N*-oxide (TMAO), a gut microbiota-derived metabolite, has become a research hotspot. Dietary phosphatidylcholine, betaine, and l-carnitine are converted by gut flora into trimethylamine, which is further metabolized by flavin monooxygenases in the liver into TMAO. There is evidence reinforcing the link between circulating TMAO levels and the increased risk of numerous non-communicable diseases such as atherosclerotic heart diseases, chronic kidney disease (CKD), and type II diabetes mellitus (DM).^8,10^ Although the role of TMAO in CKD is well documented, a precise understanding of TMAO involvement in the development of β-TM-related renal insults has yet to be researched.

Short-chain fatty acids (SCFAs) are straight-chain saturated fatty acids with less than six carbon atoms. They include acetate, propionate, butyrate, and valproic acid, which are all generated as end products of the bacterial fermentation of soluble dietary fibers in the distal small intestine and colon. SCFAs have recently emerged as crucial communicators between the gut, liver, heart, brain, and kidney and as a breakthrough for the prevention and treatment of numerous diseases.^11^ They exert antioxidant, and anti-inflammatory effects, attributed to G protein-coupled receptors, GPR41 and GPR43, activation and their histone deacetylase inhibitory activity.^11,12^ The role of SCFAs in renal diseases has been an exciting research area in recent years; however, clinical trials and laboratory studies documenting the impact of iron overload on SCFAs and linking SCFAs to β-TM-related renal insults are still lacking.

Over the years, high mobility group box 1 (HMGB1), a non-histone nuclear protein, has gained researchers’ attention as a location-dependent multifunctional biomolecule. Being actively or passively released to the extracellular compartment, it functions as a damage-associated molecular pattern molecule, modulating immune and inflammatory responses via the advanced glycation end-products receptor (RAGE) and toll-like receptors.^13^ Its release is fine-tuned chiefly by its post-translational modifications, including acetylation and deacetylation. This deeply embroils sirtuin 1 (SIRT1), a NAD^+^-dependent deacetylase, in the scene.^14^ HMGB1 is involved in the pathogenesis of numerous immune-mediated and inflammatory disorders.^13^ Of note, previous studies reported that TMAO is a regulator of SIRT1 transcriptional machinery.^15,16^ However, the potential link between gut dysbiosis, HMGB1, and its implication in β-TM-associated renal insults is not fully declared yet.

Therefore, our study aimed to evaluate the potential link between iron overload, gut leakage/dysbiosis, and kidney dysfunction in children with β-TM. We also aimed to highlight the role of the TMAO-dependent SIRT1/HMGB1 axis in mediating these relationships.

## Subjects and methods

The present work was conducted at the Medical Biochemistry, Clinical Pathology, and Pediatrics departments, Faculty of Medicine, Tanta University, during the period from June 2023 to August 2023. Forty children with β-TM aged between 6 and 18 years were included as the patient group. Forty healthy children of matched age and sex served as the control group. Written informed consent was signed by the parents of all included children. Moreover, verbal assent for research participation was obtained from all included children. The research was approved by the Local Ethics Committee of our Faculty of Medicine (code; 36264PR238/6/23). All procedures were conducted in accordance with the Declaration of Helsinki II.

### Inclusion criteria

Children with β-TM aged between 6 and 18 years.

### Exclusion criteria

Newly diagnosed β-TM children, children with a prior history of vesicoureteral reflux, hydronephrosis, recurrent urinary tract infection, renal stones, children under the umbrella of nephrotoxic medications or antibiotics, children with a family history of hereditable kidney diseases, children who were positive for viral hepatitis B or C infections or complaining of any other active infection, especially intestinal infection, immune-mediated disease, and who underwent bone marrow transplantation were excluded. Moreover, children with other hemoglobinopathies, glucose-6-phosphate dehydrogenase deficiency, or any other concurrent disease that could influence renal function, such as DM, hypertension, valvular heart diseases, hepatic diseases, and parathyroid disorders, were also excluded.

All the included children were subjected to:

1. Precise history taking focusing on the age of diagnosis, the age at the first blood transfusion session, the transfusion frequency, and the prescribed iron chelation therapy.
2. A comprehensive clinical examination was conducted with the anthropometric measurements, such as weight, height, and body mass index (BMI). BMI was estimated utilizing the consecutive formula: BMI = body weight/square height (kg/m^2^).^17^

### Blood sampling

Venous blood samples were withdrawn under complete aseptic precautions from all participants. For the β-TM group, samples were collected prior to the transfusion therapy. The specimens were split into two fractions: the first fraction was collected in ethylene diamine tetra acetic acid (EDTA)-treated tubes for hematological and molecular assays. The second fraction was moved into a sterile plain centrifuge tube, let to be clotted at room temperature, and, immediately, centrifuged at a speed of 1000 × *g* for 15 min. Sera were, in turn, isolated, aliquoted, and maintained at −20 °C until used for the biochemical studies.

### Urine sampling

First morning void specimens were obtained from all participants and immediately centrifuged at 3000 rpm for 20 min. The supernatants were then separated, aliquoted, and kept at −20 °C until use.

### Stool sampling

Fecal samples were collected and immediately transported to our laboratory for processing within 30 min. First, an aliquot of 500 µL of saturated sodium chloride solution was added to 50 mg of the dried specimens, which were allowed to sit for half an hour at room temperature. Second, the mixture was well homogenized for 3 min utilizing a high-speed homogenizer. Then, an aliquot of 20 µL of sulfuric acid (H_2_SO_4_, 10%, v/v) was added, and the combination was vortexed for 30 s. In turn, 800 µL of anhydrous ether was added, and samples were centrifuged at 4 °C for 10 min at 10,000 × *g*. The supernatants were isolated and kept frozen at −20 °C until use.^18^

3. Assessment of hematological parameters
  A complete blood count was assessed using the ADVIA 120 automated cell counter. Additionally, serum ferritin level was measured using a commercial kit employing the sandwich enzyme-linked immunosorbent assay (ELISA) procedure (MyBiosource, Inc., San Diego, CA; Cat# MBS704419).
4. Assessment of gut leakage and dysbiosis biomarkers
  Serum TMAO levels were estimated according to the protocol validated by Wekell and Barnett^19^ using equimolar concentrations of ferrous sulfate and disodium EDTA. The OD was recorded at 410 nm in the semiautomatic BTS‐350 Biosystems spectrophotometer. Moreover, fecal SCFAs were defined utilizing a microplate assay kit stocked from Fine Biotech Co., Ltd., Wuhan, China (Cat#: EH5057).
5. Assessment of oxidative and inflammatory damage
  Serum malondialdehyde (MDA) level was assessed by a previously recommended colorimetric technique,^20^ and the antioxidant milieu was evaluated by total antioxidant capacity (TAC) determination utilizing a colorimetric commercial kit (Biodiagnostic, Egypt). Inflammatory markers such as serum interleukin 8 (IL-8) and tumor necrosis factor-alpha (TNF-α) were evaluated utilizing commercial ELISA kits (Invitrogen; Thermo Fisher Scientific, Inc.; Cat# KHC0081 and KHC3011, respectively).
6. Assessment of the SIRT1/HMGB1 signaling axis
  Serum SIRT1 and HMGB1 levels were assessed using a commercial kit applying the sandwich ELISA technique (MyBiosource, Inc., San Diego, CA; Cat# MBS2021704 and MBS3804267, respectively).
7. Assessment of renal function-related biomarkers
  Serum and urinary creatinine and serum urea levels were estimated using commercial colorimetric kits (Spinreact, Girona, Spain). The estimated glomerular filtration rate (eGFR) was estimated using the modified Schwartz formula for children: [eGFR (ml/min/1.73 m^2^) = height (cm) × 0.55/S. Cr (mg/dl)].^21^ Urinary kidney dysfunction molecule-1 (KIM-1), urinary heat shock protein 72 (HSP-72), and urinary clusterin (CLU) levels were estimated with commercial ELISA kits employing the sandwich technique (MyBiosource, Inc., San Diego, CA; Cat # MBS2020304, MBS766163, and MBS046795, respectively). Moreover, urinary Ca^+^ and albumin levels were colorimetrically assayed by commercial kits ordered from Elabscience Co. (Cat # E-BC-K103-M, E-BC-K057-M, respectively).
8. Polymerase chain reaction (PCR)
  Quantitative analysis of *HSP-72*, *CLU*, and the ferroptosis-related gene, acyl-CoA synthetase long-chain family member 4 (*ACSL4*) relative gene expression was conducted by quantitative real-time polymerase chain reaction (qRT-PCR) according to the following protocol:
  1. Polymorph nuclear leukocytes (PMNLs) isolation: PMNLs were prepared via density gradient centrifugation using Ficoll-Hypaque. Briefly, EDTA blood samples were carefully layered on Ficoll and PMNLs were harvested from the white interphase after centrifugation for 15 min at 800 × *g* at room temperature and washed with phosphate-buffered saline.^22^
  2. Total RNA extraction: RNA was isolated from the PMNLS using TRIzol (Invitrogen Life Technology, Carlsbad, CA) in coherence with the provided guidelines. The concentration and quality of the isolated RNA were evaluated by monitoring OD 260 and the ratio of OD 260/280, respectively, using a NanoDrop spectrophotometer (NanoDrop Technologies, Inc., Wilmington). RNA was, in turn, maintained at −80 °C until used.
  3. Complementary DNA (cDNA) synthesis: following the provided protocol, the isolated RNA was reverse transcribed into cDNA using RevertAid H Minus Reverse Transcriptase (Thermo Scientific, MA). In turn, the cDNA was then kept at −20 °C until use.
  4. Gene amplification by qRT-PCR: the cDNA was used as a template to quantify the relative *HSP-72*, *CLU*, and *ACSL4* mRNA expression, utilizing Maxima SYBR Green/ROX Master Mix (Thermo Scientific, MA) in StepOnePlus qRT-PCR system (Applied Biosystem, CA).

The thermal cycling followed the consequent protocol: initial denaturation at 95 °C for 10 min with subsequent (40–45) cycles of (DNA denaturation at 95 °C for 15 s, primer annealing at 60 °C for 30 s, DNA elongation at 72 °C for 30 s). The melting curve was created and analyzed by raising the temperature to 95 °C by the end of the last cycle. The cycle threshold (Ct) values were calculated for the target, and glyceraldehyde-3-phosphate dehydrogenase (*GAPDH*), the constitutive gene, with the relative expression employing the 2^−ΔΔCT^ methodology.^23^ The gene-specific primers were conceived by Primer Premier 5.0 software [http://www.premierbiosoft.com/jsp/customer/RequestEvaluation.jsp], and their nucleotide sequences are depicted in Table 1.

**Table 1:** Primer sequences.

Gene	Forward primer (5′–3′)	Reverse primer (5′–3′)
*HSPA1A*	ACCAAGCAGACGCAGATCTTC	GCCCTCGTACACCTGGATCA
*CLU*	TGA TCC CAT CAC TGT GAC GG	GCT TTT TGC GGT ATT CCT GC
*ACSL4*	CCTGCAGCCATAGGTAAAGC	TTCCAAAGAGGACTCGCTG
*GAPDH*	CCACTCCTCCACCTT TGAC	ACCCTGTTGCTGTAGCCA

*HSPA1A* heat shock protein family A member 1A, *CLU* clustrin, *ACSL4* acyl-CoA synthetase long-chain family member 4, *GAPDH* glyceraldehyde-3-phosphate dehydrogenase.

### Statistical analysis

The results of the current work were presented as means and standard deviations (SD) if the data were quantitative, and as numbers and percentages if the data were categorical. Data analysis was conducted using the Statistical Package Social Sciences 20 system (SPSS Inc., Chicago, IL). The Shapiro–Wilk test was utilized to detect the normal distribution. The statistical significance between the means of the enrolled groups was determined by an independent *t*-test. Using Pearson’s correlation coefficient, a correlation was performed between clinical, hematological, and biochemical parameters. Differences were assumed to be statistically significant when the *p* value was lower than 0.05.

## Results

### Demographic and clinical characteristics of the study groups

We screened 60 patients with β-TM; 20 patients were excluded: three children were under nephrotoxic drug aminoglycoside based on culture, five children underwent hematopoietic stem cell transplantation, ten children were positive for HBV and HCV, and two children had DM with thalassemia. Forty children with β-TM met the inclusion criteria and included as the patient group and forty healthy children of matched age and sex as the control group. No statistically significant differences regarding age and sex were documented between the study groups. The mean age at first blood transfusion in the patient group was 7.9 ± 1.5 months. Weight and height were significantly lower in the patient group compared to the control group. The BMI was lower in the patient group than the control group, but it did not reach a significant level.

The clinical history of the study patients revealed that half of the patient group had splenectomy. All the β-TM children received regular blood transfusions (40% had blood transfusions every 4 weeks, 35% every 5 weeks, and 25% every 6 weeks). Regarding iron chelation therapy, 50% of the patients received deferasirox, and 50% received combined therapy in the form of a daily dose of deferiprone 75 mg/kg together with deferoxamine 25–50 mg/kg for 5–7 days a week.

Hb and hematocrit (HCT) levels were considerably lower in the patient group compared to the control group. Conversely, platelet count and serum ferritin were significantly higher in β-TM patients than in the control group (Table 2).

**Table 2:** Demographic, clinical, and laboratory characteristics of the study groups.

Parameters	Patient group	Control group	*p* value
Age (years)	13.06 ± 3.03	12.10 ± 2.95	0.15
Sex (male:female)	18:22	20:20	0.34
Weight (kg)	32.30 ± 4.60	35.40 ± 6.34	0.01
Weight *Z* score	0.00 ± 0.99	0.00 ± 0.99	1.00
Height (cm)	135.90 ± 6.49	142.40 ± 6.02	<0.001
Height *z* score	0.10 ± 1.00	0.10 ± 0.99	0.98
BMI (kg/m^2^)	17.37 ± 1.15	17.52 ± 3.03	0.79
BMI *Z* score	0.00 ± 1.00	0.01 ± 1.17	0.96
History of splenectomy	50%	0%	–
History of regular blood transfusion			
Every 4 weeks (%)	40%	0%	–
Every 5 weeks (%)	35%	0%	–
Every 6 weeks (%)	25%	0%	–
Iron chelation therapy			
Deferasirox (%)	50%	0%	–
Combined (%)	50%	0%	–
Hb (g/dl)	6.77 ± 0.41	11.95 ± 0.33	<0.001
HCT (%)	20.19 ± 1.21	35.83 ± 0.93	<0.001
Platelet count (×10^3^ cell/μl)	483.87 ± 71.26	285.50 ± 63.06	<0.001
Serum ferritin (ng/ml)	2203.12 ± 378.77	55.90 ± 20.61	<0.001
Age at first blood transfusion (m)	7.98 ± 1.51	–	–

Continuous variables were presented as mean ± standard deviation (SD).

*Hb* hemoglobin, *HCT* hematocrit, *BMI* body mass index.

### Renal function biomarkers

As depicted in Table 3, eGFR was substantially lower in the patient group than in the control group. Serum urea, creatinine, urinary Ca^+^/Cr ratio, urinary Alb/Cr ratio, and urinary KIM-1/Cr were significantly elevated in thalassemic children than in the control group. Moreover, patients’ urinary levels of HSP-72 and CLU were significantly higher than those in the control group.

**Table 3:** Biomarkers of kidney function and kidney injury in study groups.

Parameters	Patient group	Control group	*P* value
Serum creatinine (mg/dl)	0.59 ± 0.09	0.52 ± 0.09	0.001
Serum urea (mg/dl)	16.12 ± 2.08	13.70 ± 1.20	<0.001
eGFR (ml/min/1.73 m^2^)	131.78 ± 14.89	154.32 ± 24.27	<0.001
Urinary Ca/Cr (mg/gm)	191.72 ± 52.89	158.20 ± 11.88	<0.001
Urinary Alb/Cr (mg/gm)	47.30 ± 30.12	22.68 ± 3.46	<0.001
Urinary KIM-1/Cr (ng/mg Cr)	5.85 ± 0.74	0.72 ± 0.16	<0.001
Urinary HSP-72 level (pg/ml)	34.26 ± 4.05	18.20 ± 1.57	<0.001
Urinary CLU level (µg/ml)	747.26 ± 79.04	310.37 ± 36.02	<0.001

Continuous variables were presented as mean ± standard deviation (SD).

*eGFR* estimated glomerular filtration rate, *Ca/Cr* calcium to creatinine ratio, *Alb/Cr* albumin to creatinine ratio, *KIM/Cr* kidney injury molecule to creatinine ratio, *HSP-72* heat shock protein 72, *CLU* clustrin.

### Oxidative stress and inflammation biomarkers

Redox imbalance appeared as a fundamental hallmark in thalassemic patients, denoted by the significant elevation of MDA level, a biomarker for lipid peroxidation, combined with the significant decline of TAC in the patient group compared to the control group. Furthermore, the serum concentrations of the pro-inflammatory cytokines, TNF-α and IL-8, were significantly higher in the patient group compared to the control group (Table 4).

**Table 4:** Oxidative stress and inflammation biomarkers among the study groups.

Parameters	Patient group	Control group	*p* value
MDA (umol/L)	10.77 ± 1.00	1.79 ± 0.16	<0.001
TAC (mmol/L)	1.72 ± 0.35	3.23 ± 0.76	<0.001
IL-8 (pg/ml)	255.50 ± 30.90	100.20 ± 12.36	<0.001
TNF-α (pg/ml)	747.42 ± 71.36	165.56 ± 24.89	<0.001

Continuous variables were presented as mean ± standard deviation (SD).

*MDA* malondialdehyde, *TAC* total antioxidant capacity, *IL-8* interleukin 8, *TNF-α* tumor necrosis factor-alpha.

### Serum TMAO, SIRT1, HMGB1, and fecal SCFAs levels

**Fig. 1:**
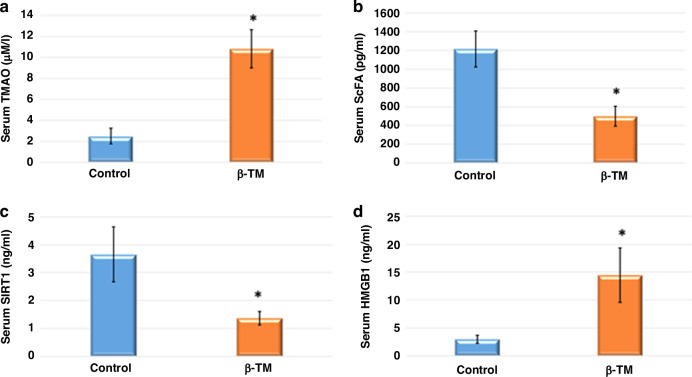
Serum TMAO, HMGB1, SIRT1, and fecal SCFAs levels in the study groups. **a** Serum TMAO trimethylamine-*N*-oxide; **b** serum HMGB1 high mobility group box 1; **c** serum SIRT1 sirtuin 1; **d** fecal SCFAs short-chain fatty acids; data presented as means ± SD. * denotes significant and *P* < 0.05, number of participants in each group = 40.

Our data revealed that serum TMAO and HMGB1 levels were significantly higher in the patient group compared to the control group. Meanwhile, the patient group displayed markedly lower serum SIRT1 levels as well as fecal SCFAs than the control group (Fig. 1).

**Fig. 2:**
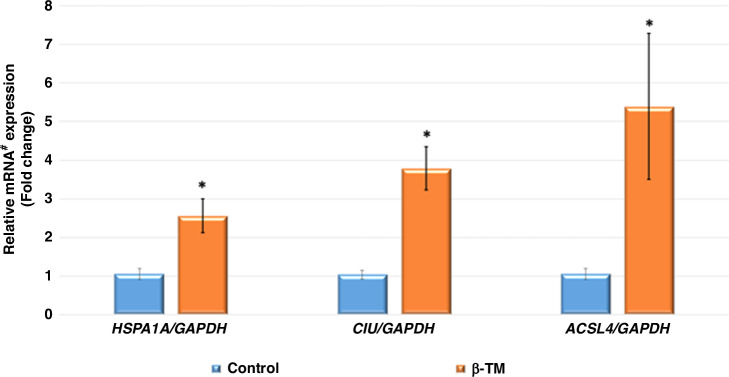
*HSPA1A*, *CLU*, and *ACSL4* messenger RNA relative expression in the study groups. Data presented as means ± SD, *P* value between groups was determined by independent *t*-test, * denotes that *P* is significant and <0.05, *HSA1A* heat shock protein family A member 1A, *CLU* clusterin, *ACSL4* acyl-CoA synthetase long-chain family member 4, PMNLs polymorphonuclear leukocytes, number of participants in each group = 40.

Figure 2 showed that relative mRNA expressions of *HSPA1A*, *CLU*, and *ACSL4* were notably upregulated in children with β-TM compared to the control group.

### Correlations between the studied parameters in the β-TM group

There was a significant positive correlation between serum ferritin and each of the urinary KIM-1/Cr ratio, urinary HSP-72, urinary CLU, serum MDA, TNF-α, serum TMAO levels, and the relative mRNA expression of *ACSL4* gene. Meanwhile, there was a significant negative correlation between serum ferritin level, and each of the fecal SCFAs and the age at the start of transfusion sessions in the β-TM group (Table 5).

**Table 5:** Correlation between serum ferritin and the studied parameters in β-TM group.

Parameters	Serum ferritin	
	*r*	*P*
Age (y)	0.71	<0.001
Age at first transfusion (months)	−0.82	<0.001
Serum TMAO	0.83	<0.001
Urinary KIM-1/Cr (ng/mg Cr)	0.74	<0.001
Urinary HSP-72 (pg/ml)	0.78	<0.001
Urinary CLU (µg/ml)	0.86	<0.001
MDA (umol/L)	0.77	<0.001
TNF-α (pg/ml)	0.88	<0.001
Fecal SCFAs (pg/ml)	−0.77	<0.001
ACSL4 fold change	0.823	<0.001

*TMAO* trimethylamine-*N*-oxide, *KIM-1/Cr* kidney injury molecule-1/creatinine ratio, *HSP-72* heat shock protein 72, *CLU* clusterin, *MDA* malondialdehyde, *TNF-α* tumor necrosis factor-alpha, *SCFAs* short-chain fatty acids, *ACSL4* acyl-CoA synthetase long-chain family member 4.

Interestingly, there was a significant positive correlation between serum TMAO level and each of serum ferritin, serum HMGB1, urinary KIM-1/Cr, urinary CLU, urinary HSP-72, serum MDA, and TNF-α, as well as the relative expression of *ACSL4*. In contrast, there was a significant negative correlation between serum TMAO level and fecal SCFAs, serum SIRT1, and age at the start of transfusion in the β-TM children (Table 6).

**Table 6:** Correlation between circulating TMAO and the studied parameters in β-TM group.

Parameters	TMAO	
	*r*	*P*
Age (years)	0.86	<0.001
Age at first transfusion (months)	−0.88	<0.001
Serum ferritin (ng/ml)	0.83	<0.001
Urinary KIM-1/Cr (ng/mg Cr)	0.96	<0.001
Urinary HSP-72 (pg/ml)	0.98	<0.001
Urinary CLU (µg/ml)	0.98	<0.001
MDA (umol/L)	0.97	<0.001
TNF-α (pg/ml)	0.95	<0.001
Fecal SCFAs (pg/ml)	−0.83	<0.001
ACSL4 fold change	0.96	<0.001
SIRTI (ng/ml)	−0.96	<0.001
HMGBI (ng/ml)	0.99	<0.001

*TMAO* trimethylamine-*N*-oxide, *SIRT1* serum level of sirtuin 1, *HMGB1* serum level of high mobility group box 1, *KIM-1/Cr* kidney injury molecule-1/creatinine ratio, *HSP-72* heat shock protein 72, *CLU* urinary clusterin, *MDA* malondialdehyde, *TNF-α* tumor necrosis factor-alpha, *SCFAs* short-chain fatty acids, *ACSL4* acyl-CoA synthetase long-chain family member 4.

## Discussion

The gut microbiota has emerged as a virtual organ, producing numerous bioactive metabolites.^24^ Emerging data reveal that gut barrier defects and the aberrant composition of the gut microbiota are deeply involved in the pathogenesis of several diseases.^25^ Owing to this perspective, the present work is the first to assess the potential link between frequent blood transfusion-induced iron overload, gut leakage/dysbiosis, and the pathophysiology of subclinical renal insult in children with β-TM.

The current research, in agreement with previous studies^5,26,27^ has shown a significantly elevated serum ferritin level in the patient group in comparison to the control group, indicating the presence of iron overload. This is due to the long-standing blood transfusions coupled with the increased gastrointestinal iron absorption on top of β-TM-related ineffective erythropoiesis.^28^

The present work speculates that the altered iron homeostasis in children with β-TM could reshape the gut microbial profile and disrupt the intestinal barrier’s physiological function. This was evidenced by the profound increment of serum TMAO level, coupled with a significant decrease of the fecal SCFAs in the patient group. To the best of our knowledge, no previous studies have explored the impact of iron overload on these cardinal biomolecules. Similarly, Visitchanakun et al.^9^ reported that iron accumulation within the intestinal mucosal cells of the thalassemic mice has disrupted gut permeability. Additionally, Sivaprakasam et al.^29^ validated that hereditary hemochromatosis could induce profound changes in the colonic microbial profile in favor of the pathogenic bacteria, besides loss of the colonic barrier function.

Iron overload is one of the aspects of iron metabolism within the intestinal mucosal cells. Iron induces mucosal OS, persistent inflammation, and ferroptosis.^30^ These phenomena are critical factors that precipitate barrier damage, defective secretion of antimicrobial peptides, gut microbial profile alteration, and disruption of the entire intestinal homeostasis.^26^ In the current study, it was clearly obvious that redox homeostasis was considerably disrupted in children with β-TM, as validated by the significant elevation of the lipid peroxidation biomarker, MDA, and the simultaneous decline of the serum TAC. According to previous workers, redox imbalance provokes an inflammatory response and triggers ferroptotic cell death^.^^5,31,32^ Iron/OS/inflammation relationship was reported in thalassemic patients by Keshk et al.^5^ while iron/ferroptosis relationship was recorded in patients with hemophilic arthropathy and those with type II diabetic patients by Han et al.^31^ and Deng et al.^32^, respectively. Literature based, accumulation of membrane lipid peroxides, as indicated by MDA increment herein, leads to the execution of ferroptosis by altering iron transport across the plasma membrane.^33,34^ This is in line with our results, which demonstrated parallel induction of OS, circulating cytokines, and upregulation of the ferroptosis marker *ACSL4* in PMNLs. Accordingly, iron could be a crucial crosstalk node between OS, inflammation, and ferroptosis. To be honest, the cross-sectional nature of the work can only suggest that but not swearing on such relationship.

Besides the iron-induced compromise, earlier reports revealed that the circulating TMAO is positively associated with the intensity of oxidative and inflammatory damage and is deeply involved in cell death mechanisms.^35–37^ Our correlation results partially support this consensus and present TMAO as a potential hidden hand behind the β-TM-associated damage and a new scientific enigma. From our point of view, the unique way TMAO affects the cellular microenvironment could be attributed to its ability to reach a readily detectable circulating level and to remain relatively stable over time, unlike the other swiftly metabolized microbial byproducts.

Interestingly, the current work reported the presence of β-TM-related renal insult, validated by the significant rise in serum urea in the patient group, which came in harmony with the findings of Mahmoud et al.^38^. Moreover, our findings greatly matched the results of other researchers^39–41^ who recorded a progressive decline in eGFR among the thalassemic patients when compared to the controls. Furthermore, tubular damage markers such as urinary Ca^+^/Cr ratio and urinary Alb were significantly elevated in children with β-TM relative to normal children. In this context, our observation was consistent with previous researchers^27,42–44^ who correlated such findings with proximal tubulopathy. In coherence with other research,^45–48^ U. KIM-1/Cr ratio, U. HSP-72, and U. CLU, well-validated early and highly sensitive predictors of tubular dysfunction, exhibited significantly higher levels in children with β-TM carrying more evidence of renal dysfunction.

Several rationales were suggested to explain the pathophysiology underlying the β-TM-related renal insult, which is a long-term thalassemic complication.^5^ While our study is cross-sectional and was not designed to establish causality, several novel mechanisms of kidney dysfunction in β-TM can be proposed based on our findings. The chronic anemic state and its related hypoxic microenvironment, iron overload-induced/TMAO-augmented general state of OS, inflammation, and ferroptosis appeared to be highly implicated. Indeed, kidney function, OS, inflammation, and ferroptotic biomarkers all were strongly correlated with both serum ferritin and serum TMAO.

Besides, a thorough analysis of our data enabled us to innovatively explain the renal tubular cell damage based on the TMAO impact on SIRT1 and its dependent network of biomolecules, including HMGB1. SIRT1 has been depicted as a cardinal regulator of OS and inflammation.^15^ Indeed, this is accomplished by SIRT1 ability to deacetylate histone and numerous non-histone proteins, including nuclear factor-kappa b (NF-κB). NF-κB deacetylation results in inhibition of TNF-α expression and other pro-inflammatory cytokines.^49^ Our work identified a profound decrement in serum SIRT1 in children with β-TM, which was concomitantly correlated with a notable rise in serum OS, inflammatory, and ferroptotic markers. It was formerly stated that TMAO can directly inhibit the SIRT1 transcriptional machinery, thus abrogating SIRT1 renoprotective effects.^15,50^ However, the precise molecular pathophysiology remains to be established.

HMGB1, a DNA-binding protein, regulates chromatin accessibility. The extracellular HMGB1 can activate downstream signaling cascades that eventually end in inflammation, tissue damage, and cell death. Few studies implicated HMGB1 in renal disease initiation and progress.^51,52^ Recently, numerous studies have elucidated the close relationship between SIRT1 and HMGB1 and illustrated that SIRT1 and HMGB1 signaling pathways have an antagonistic effect. It has been stated that HMGB1 is continuously maintained under tight SIRT1-dependent control mechanisms.^53^ First, both directly interact, forming a cellular stable complex.^54^ Second, HMGB1, being a SIRT1 substrate, is deacetylated and kept in the nucleus. Furthermore, SIRT1 can control the matter from the roots by inhibiting HMGB1 gene transcription.^53^ The present work has ensured this antagonistic relationship, and the conducted correlation studies strongly linked the deranged SIRT1/HMGB1 functional axis to TMAO levels. In fact, TMAO could reduce SIRT1 in the thalassemic group, allowing feasible export of HMGB1 to the extracellular compartment, binding to its bio-specific receptors, and promoting multi-organ damage, including the kidney. Shedding light on this speculation could inspire innovative research in such a hot spot, allowing the development of new predictive, preventive, and therapeutic modalities for such patients.

If our hypothesis is proven to be correct, we can say that correction of gut dysbiosis would slow down the progression of kidney dysfunction in β-TM patients. Moreover, we can call for an initiative to launch TMAO-targeting medications and include them in the treatment protocol of these patients.

### Limitations of the study

The cross-sectional nature of this work does not allow for establishing causality that presents a major study limitation. Moreover, the use of creatinine-based estimated GFR as a marker of kidney function in children with this primarily tubular rather than glomerular kidney dysfunction is another limitation of the study. Also, we used granulocytes as surrogates for tubular cells when assessing ferroptosis. To ensure the validity of our proposal and to relieve any existing confoundings, we aim to conduct a future cohort study on a group of newly diagnosed β-TM children and parallelly monitor changes in each of TMAO and kidney function biomarkers, including eGFR to determine which of gut dysbiosis or impaired eGFR is the actual cause beyond the β-TM-related renal dysfunction.

## Conclusion

Our study suggests a link between iron overload, gut dysbiosis/leakage, and kidney dysfunction in children with β-TM. TMAO, a gut leakage/dysbiosis biomarker, may have a role in the pathogenesis of β-TM-related subclinical renal tubular dysfunction by exacerbating iron-initiated OS, inflammation, ferroptosis, and modulating the TMAO-dependent SIRT1/HMGB1 axis.

## Data Availability

The data support the findings of this study are available on request from the corresponding author.
